# Supplementary material for: Reconstitution of BNIP3/NIX-mediated autophagy reveals two pathways and hierarchical flexibility of the initiation machinery
Source: bioRxiv. 2024 Aug 28:2024.08.28.609967. Preprint. [Version 1] doi: 10.1101/2024.08.28.609967 (PMC11383309; doi:10.1101/2024.08.28.609967)
Supplement: Supplement 1 [file NIHPP2024.08.28.609967v1-supplement-1.pdf]

bioRxiv preprint doi: https://doi.org/10.1101/2024.08.28.609967; this version posted August 28, 2024. The copyright holder for this preprint (which was not certified by peer review) is the author/funder, who has granted bioRxiv a license to display the preprint in perpetuity. It is made available under aCC-BY 4.0 International license.

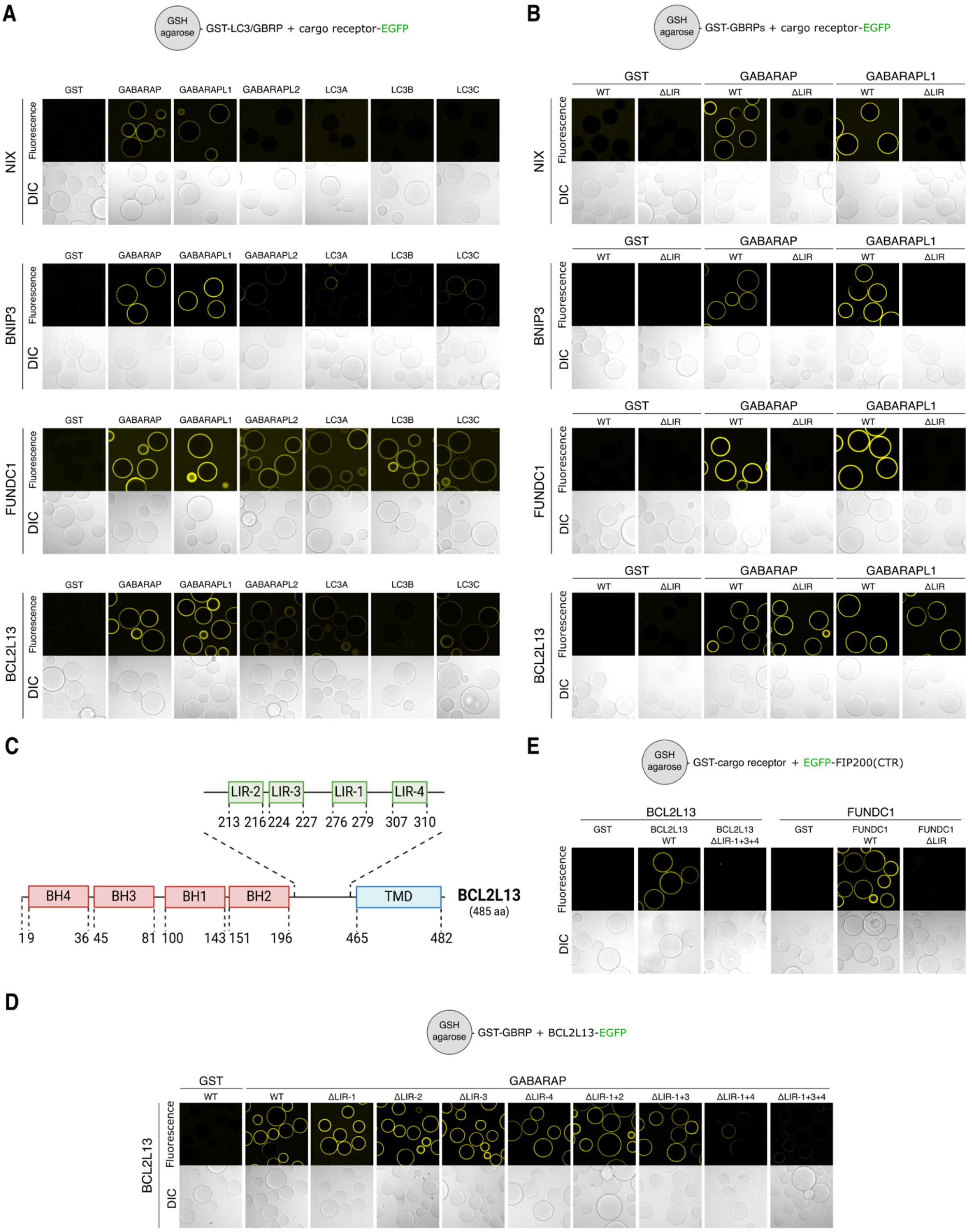

Figure S1. In vitro validation of mitophagy cargo receptors and their LIR/FIR motifs
(**A**) Microscopy-based bead assay of agarose beads coated with GST-tagged LC3A/B/C or GBRP/GBRPL1/GBRPL2 and incubated with GFP-tagged cargo receptors FUNDC1, BCL2L13, NIX, and BNIP3. (**B**) As in (A) but with wild-type (WT) or alanine-mutated LIR-motifs (ΔLIR) of the GFP-tagged cargo receptors. (**C**) Schematic of domain structure of BCL2L13 with the candidate LIR/FIR motifs indicated with residue numbers. LIR-1 was previously annotated in literature as the active LIR motif. (**D**) As in (A), but with different alanine-mutated

bioRxiv preprint doi: https://doi.org/10.1101/2024.08.28.609967; this version posted August 28, 2024. The copyright holder for this preprint (which was not certified by peer review) is the author/funder, who has granted bioRxiv a license to display the preprint in perpetuity. It is made available under aCC-BY 4.0 International license.

variants of the different LIR-motifs (ΔLIR) of GFP-tagged BCL2L13. (**E**) As in (A) but with GST-tagged cargo receptors and GFP-tagged C-terminal region (CTR; 1429-1591aa) of FIP200.

bioRxiv preprint doi: https://doi.org/10.1101/2024.08.28.609967; this version posted August 28, 2024. The copyright holder for this preprint (which was not certified by peer review) is the author/funder, who has granted bioRxiv a license to display the preprint in perpetuity. It is made available under aCC-BY 4.0 International license.

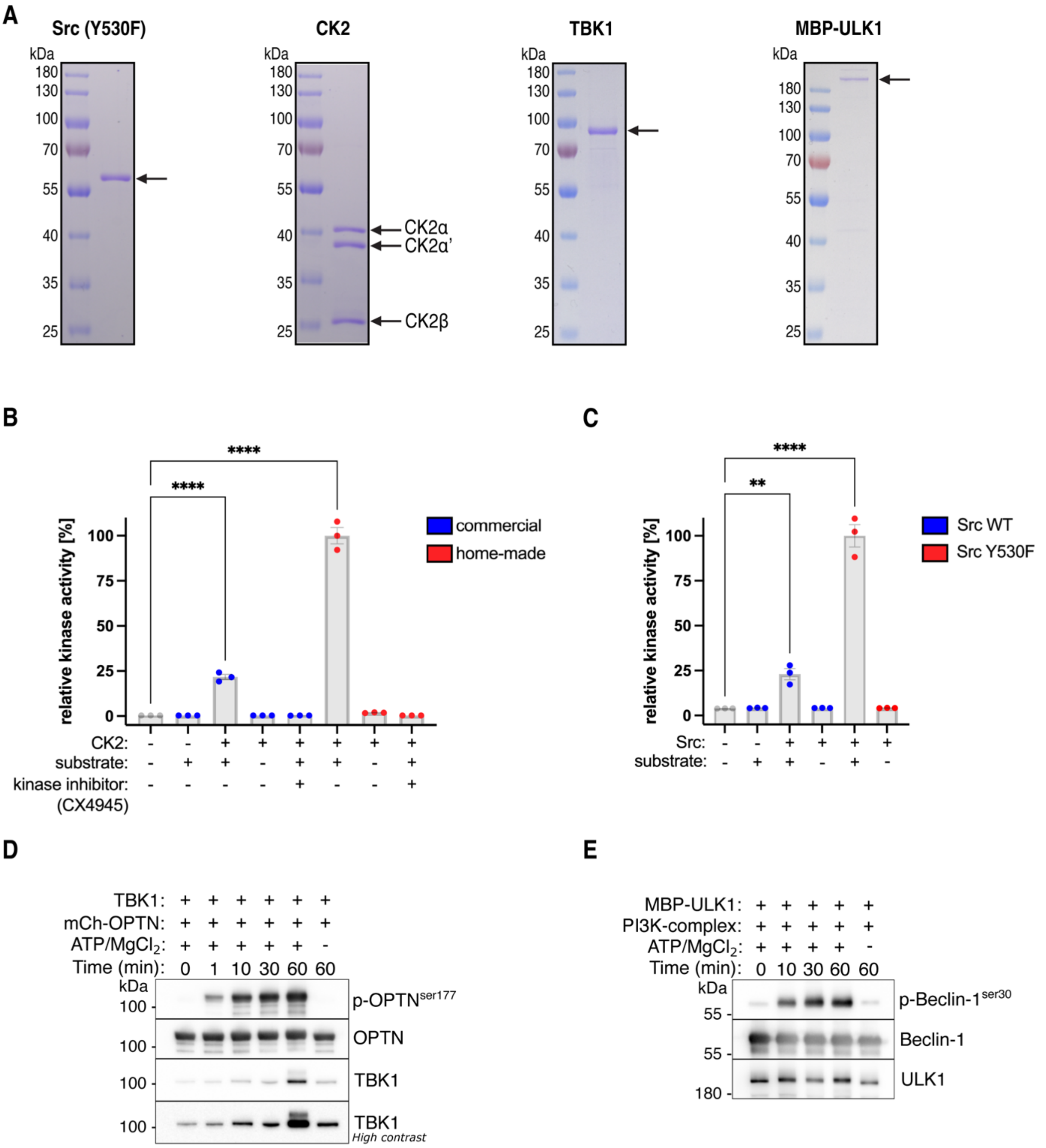

Figure S2. Purified kinases and validation of their activity
(**A**) Representative SDS-PAGE gels of purified Src (Y530F), CK2 complex, TBK1, and MBP-ULK1. Arrows indicate the predicted molecular weight. (**B-C**) Measurement of kinase activity using a plate-reader based read-out. Kinases were incubated with or without a substrate peptide or kinase inhibitor. Kinase activity was compared between our purified CK2 complex (home-made) and commercially available CK2, or between wild-type (WT) and Y530F mutant Src. (**D**) Measurement of kinase activity by mixing recombinantly purified mCherry-OPTN and TBK1 for the indicated time and western blot analysis using antibodies for phosphorylated OPTN (S177) as a read out for TBK1 activity. (**E**) As in D, but after mixing recombinantly purified MBP-ULK1 and the PI3KC3-C1 complex (composed of ATG14, Beclin-1, Vps15, Vps34) for the indicated time and using antibodies for phosphorylated Beclin-1 (Ser30) as a

bioRxiv preprint doi: https://doi.org/10.1101/2024.08.28.609967; this version posted August 28, 2024. The copyright holder for this preprint (which was not certified by peer review) is the author/funder, who has granted bioRxiv a license to display the preprint in perpetuity. It is made available under aCC-BY 4.0 International license.

read out for ULK1 activity. One-way ANOVA with Dunnett's multiple comparisons test (B, C). **P<0.005, ****P<0.0001. ns, not significant.

bioRxiv preprint doi: https://doi.org/10.1101/2024.08.28.609967; this version posted August 28, 2024. The copyright holder for this preprint (which was not certified by peer review) is the author/funder, who has granted bioRxiv a license to display the preprint in perpetuity. It is made available under aCC-BY 4.0 International license.

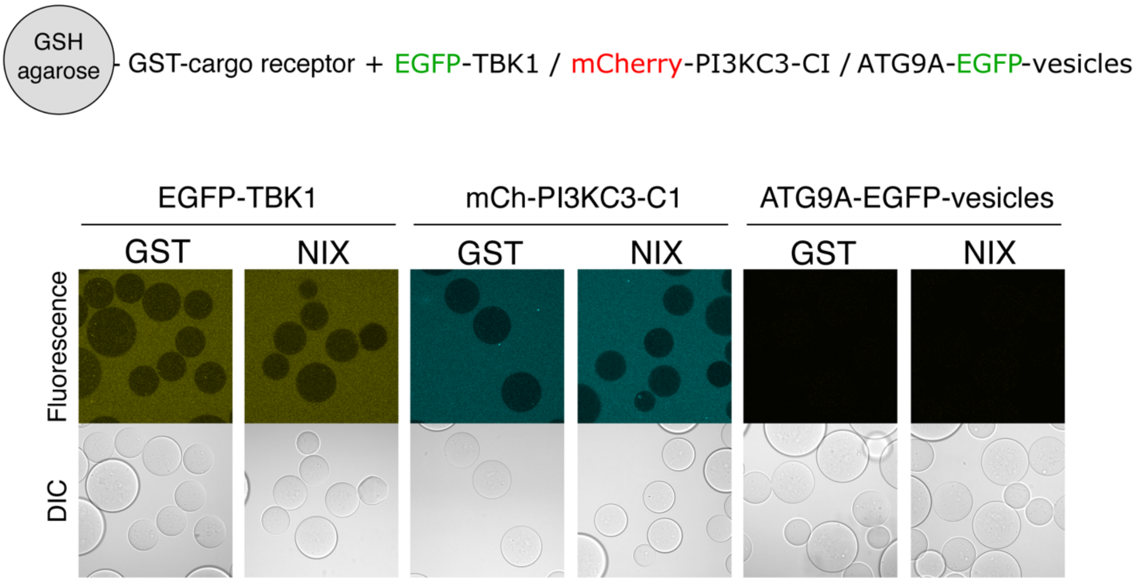

Figure S3. NIX does not interact with TBK1, PI3KC3-C1 complex, or purified ATG9A-vesicles

Microscopy-based bead assay of agarose beads coated with GST-tagged NIX and incubated with GFP-tagged TBK1, mCherry-tagged PI3KC3-C1, or GFP-tagged ATG9A-vesicles purified from HAP1 cells. GST served as negative control.

bioRxiv preprint doi: https://doi.org/10.1101/2024.08.28.609967; this version posted August 28, 2024. The copyright holder for this preprint (which was not certified by peer review) is the author/funder, who has granted bioRxiv a license to display the preprint in perpetuity. It is made available under aCC-BY 4.0 International license.

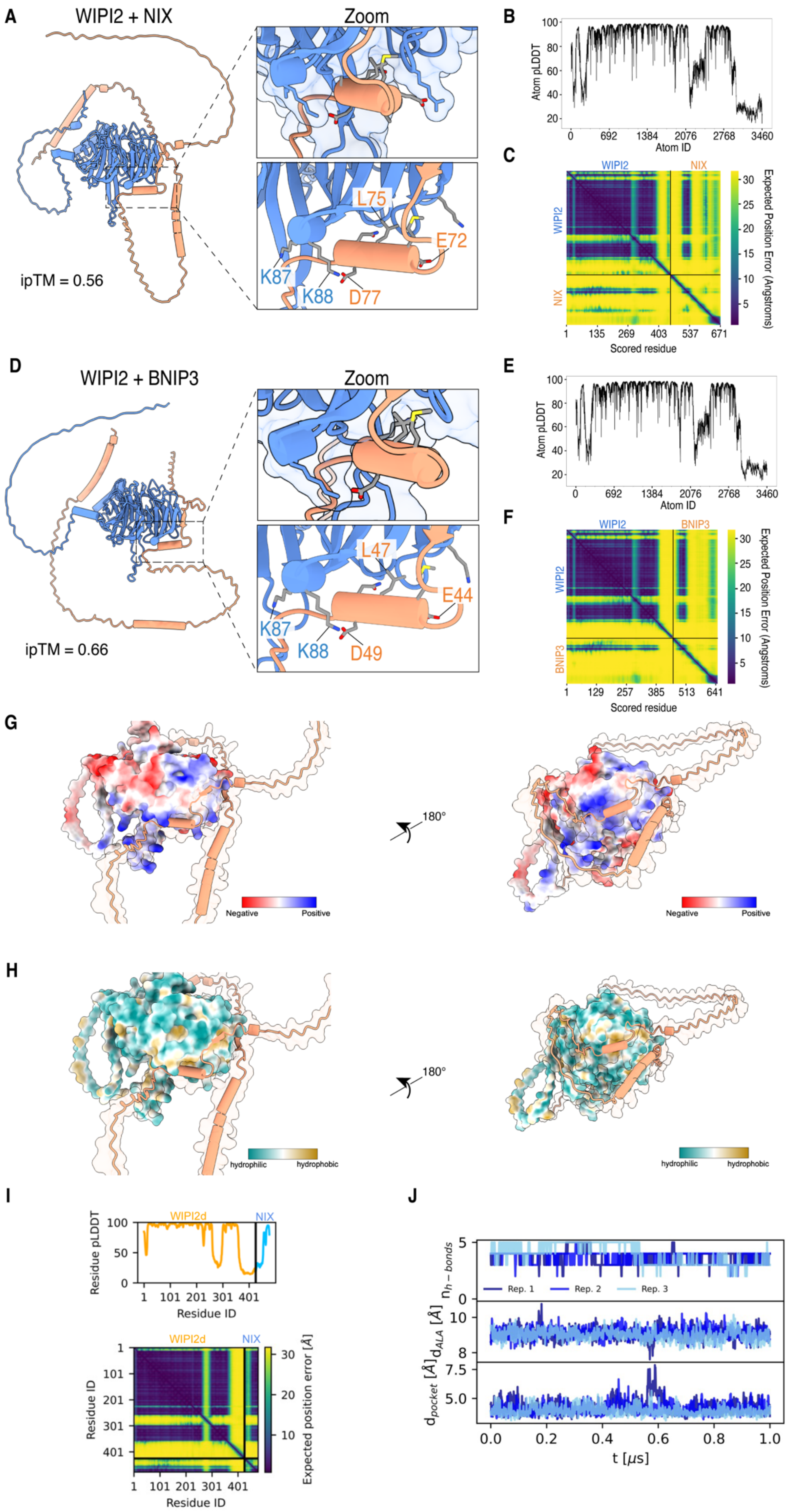

bioRxiv preprint doi: https://doi.org/10.1101/2024.08.28.609967; this version posted August 28, 2024. The copyright holder for this preprint (which was not certified by peer review) is the author/funder, who has granted bioRxiv a license to display the preprint in perpetuity. It is made available under aCC-BY 4.0 International license.

Figure S4. AlphaFold-2 prediction and MD simulations of BNIP3/NIX-WIPI2 complex
(**A**) AlphaFold-2 predicted structure of NIX (orange) and WIPI2 (blue) with zoom in on the interaction interface. (**B-C**) pLDDT and PAE plots for NIX-WIPI2 structure. (**D**) AlphaFold-2 predicted structure of BNIP3 (orange) and WIPI2 (blue) with zoom in on the interaction interface. (**E-F**) pLDDT and PAE plots for BNIP3-WIPI2 structure. (**G**) Predicted structure for the NIX-WIPI2 complex with the surface of WIPI2 colored based on electrostatics. (**H**) Predicted structure for the NIX-WIPI2 complex with the surface of WIPI2 colored based on hydrophobics. Note that the indicated residue numbers for WIPI2 correspond to their residue number in the WIPI2d sequence (which match residue numbers K105 and K106 in WIPI2b). (**I**) Residue pLDDT and PAE scores for the prediction in Fig. 2i. (**J**) The NIX W36A/L39A (ΔLIR) mutant does not bind the cryptic pocket of WIPI2d. Number of backbone h-bonds $n_{h\text{-}bonds}$ between the LIR of NIX and WIPI2d, insertion depth d_{ALA} of NIX ΔLIR A36, and minimum heavy atom distance d_{pocket} between WIPI2d F169 and I133 from three 1 μs MD simulations.

bioRxiv preprint doi: https://doi.org/10.1101/2024.08.28.609967; this version posted August 28, 2024. The copyright holder for this preprint (which was not certified by peer review) is the author/funder, who has granted bioRxiv a license to display the preprint in perpetuity. It is made available under aCC-BY 4.0 International license.

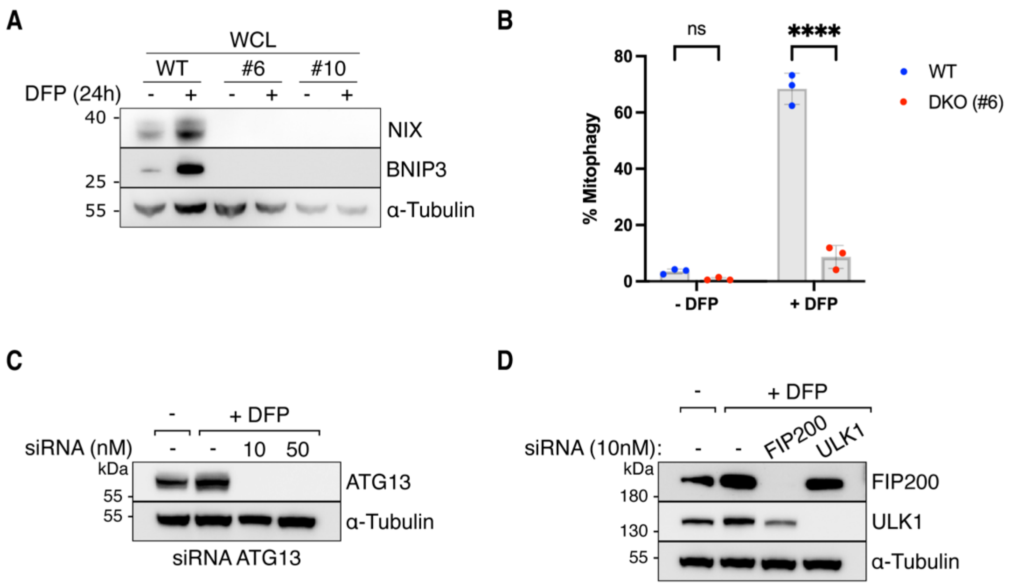

Figure S5. Validation knockout and knockdown cell lines
(**A**) Analysis of whole cell lysates (WCL) by SDS-PAGE and western blotting for NIX/BNIP3 double knockout clones #6 and #10, with and without induction of mitophagy by 24 h of DFP treatment. (**B**) Mitophagy flux was measured by flow cytometry of wild-type (WT) or NIX/BNIP3 double knockout (DKO) HeLa cells (clone #6), left untreated or treated with DFP for 24 h. (**C**) Analysis of knockdown efficiency for ATG13. HeLa cells were transfected 72 h prior to the FACS experiment, treated with DFP for 24 h to induce mitophagy, and analyzed by flow cytometry. Cells were collected after the experiment and analyzed by SDS-PAGE and western blotting. The concentration of 10 nM was used for the FACS experiment represented in the manuscript. (**D**) As in (C), but for HeLa cells transfected with siRNAs against FIP200, ULK1 or scrambled as a control (-). Two-way ANOVA with Tukey's multiple comparisons test. ****P<0.0001. ns, not significant.

bioRxiv preprint doi: https://doi.org/10.1101/2024.08.28.609967; this version posted August 28, 2024. The copyright holder for this preprint (which was not certified by peer review) is the author/funder, who has granted bioRxiv a license to display the preprint in perpetuity. It is made available under aCC-BY 4.0 International license.

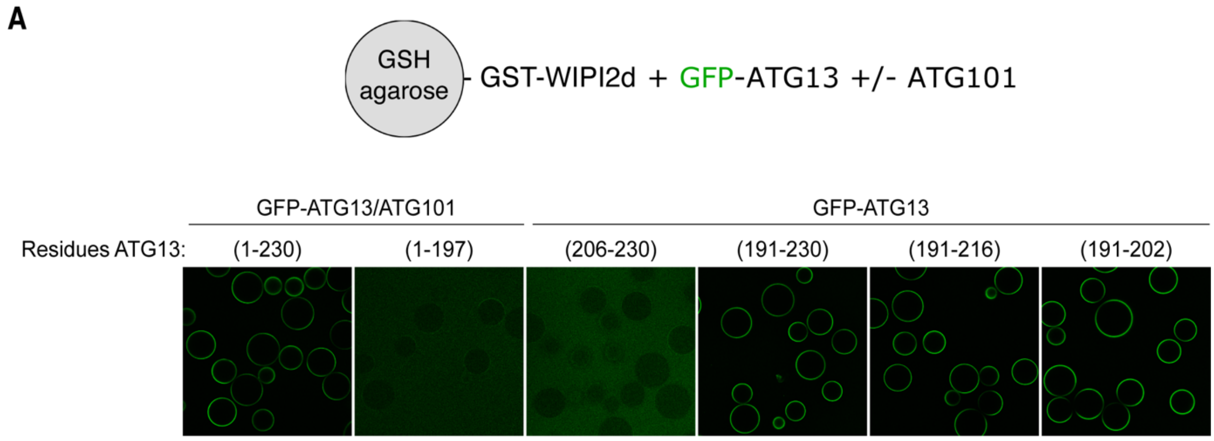

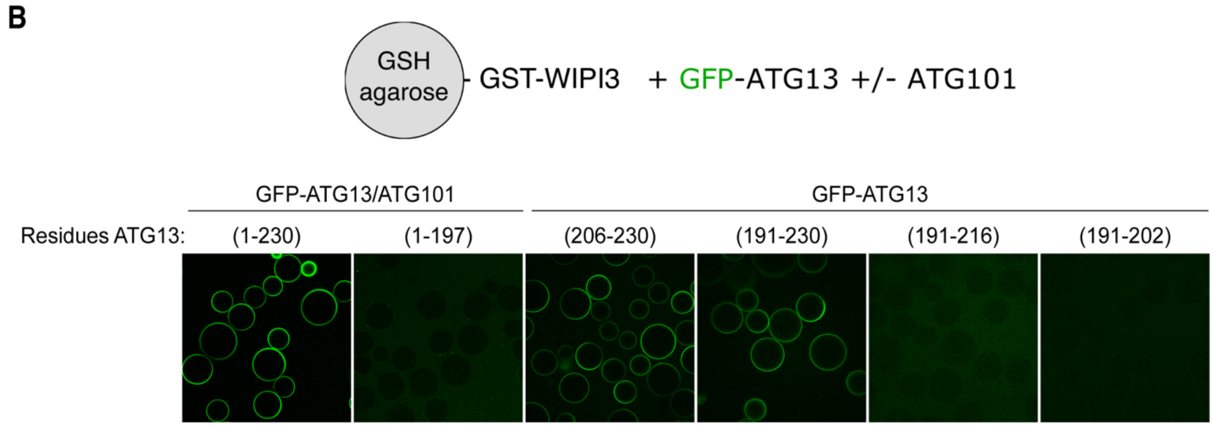

Figure S6. Biochemical mapping of binding sites of WIPI-ATG13 interaction
Microscopy-based bead assay of agarose beads coated with GST-tagged (**A**) WIPI2d or (**B**) WIPI3 and incubated with GFP-tagged ATG13/ATG101 subcomplex or fragments of ATG13 alone.

bioRxiv preprint doi: https://doi.org/10.1101/2024.08.28.609967; this version posted August 28, 2024. The copyright holder for this preprint (which was not certified by peer review) is the author/funder, who has granted bioRxiv a license to display the preprint in perpetuity. It is made available under aCC-BY 4.0 International license.

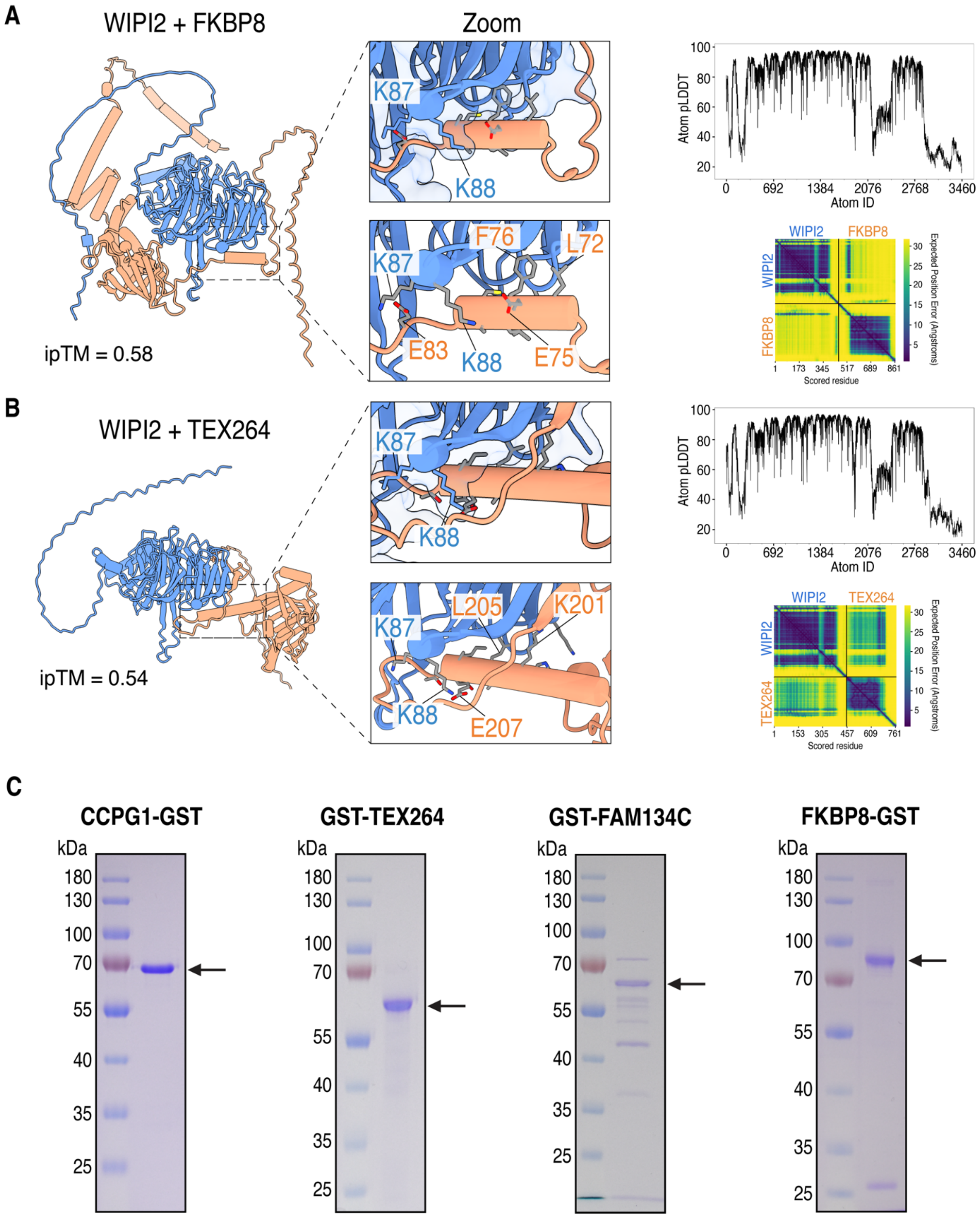

Figure S7. AlphaFold-2 prediction of WIPI2d and transmembrane cargo receptors
(**A-B**) AlphaFold-3 predicted structure for WIPI2 with (A) TEX264, or (B) FKBP8, with zoom in on the interaction interface. Note that the indicated residue numbers for WIPI2 correspond to their residue number in the WIPI2d sequence (which match residue numbers K105 and K106 in WIPI2b). pLDDT plots and predicted alignment error (PAE) heatmap are also shown. (**C**) Representative SDS-PAGE gels stained with Coomassie Brilliant Blue of purified CCPG1(1-212aa)-GST, GST-TEX264(28-313aa), GST-FAM134C(250-466aa), and FKBP8(1-391aa)-GST. Arrows indicate the predicted molecular weight.
